# Retraction Note: Topical bismuth oxide-manganese composite nanospheres alleviate atopic dermatitis-like inflammation

**DOI:** 10.1186/s12951-024-02692-1

**Published:** 2024-07-05

**Authors:** Mengjie Li, Benjin Chen, Lingling Xu, Yu Wang, Zhu Chen, Bingyan Ma, Shichun Qin, Yechun Jiang, Cheng Gu, Haisheng Qian, Fengli Xiao

**Affiliations:** 1 Department of Dermatology of First Affiliated Hospital, and Institute of Dermatology, Anhui Medical University, Hefei, 230032 Anhui China; 2 Key Laboratory of Dermatology, Anhui Medical University, Ministry of Education, Hefei, Anhui China; 3 The Center for Scientific Research of Anhui Medical University, Hefei, Anhui China; 4 School of Biomedical Engineering, Research and Engineering Center of Biomedical Materials, Anhui Provincial Institute of Translational Medicine, Anhui Medical University, Hefei, 230032 Anhui China; 5 Inflammation and Immune Mediated Diseases Laboratory of Anhui Province, Hefei, China

**Retraction Note: Journal of Nanobiotechnology (2023) 21:430**

10.1186/s12951-023-02207-4

The authors have retracted this article. After publication, the authors found that a number of images in Figs. 5, 6 and 7 were presented incorrectly, which may affect the readers’ interpretation of these results.

All authors agree to this retraction.

